# 4EBP-Dependent Signaling Supports West Nile Virus Growth and Protein Expression

**DOI:** 10.3390/v8100287

**Published:** 2016-10-18

**Authors:** Katherine D. Shives, Aaron R. Massey, Nicholas A. May, Thomas E. Morrison, J. David Beckham

**Affiliations:** 1 Department of Immunology and Microbiology, University of Colorado Graduate School, Aurora, CO 80045, USA; katherineshives@gmail.com (K.D.S.); nicholas.may@ucdenver.edu (N.A.M.); thomas.morrison@ucdenver.edu (T.E.M.); 2 Department of Medicine, Division of Infectious Diseases, University of Colorado School of Medicine, Aurora, CO 80045, USA; aaron.massey@ucdenver.edu

**Keywords:** West Nile virus, RNA, translation, protein synthesis

## Abstract

West Nile virus (WNV) is a (+) sense, single-stranded RNA virus in the *Flavivirus* genus. WNV RNA possesses an ^m7^GpppN_m_ 5′ cap with 2′-*O*-methylation that mimics host mRNAs preventing innate immune detection and allowing the virus to translate its RNA genome through the utilization of cap-dependent translation initiation effectors in a wide variety of host species. Our prior work established the requirement of the host mammalian target of rapamycin complex 1 (mTORC1) for optimal WNV growth and protein expression; yet, the roles of the downstream effectors of mTORC1 in WNV translation are unknown. In this study, we utilize gene deletion mutants in the ribosomal protein kinase called S6 kinase (S6K) and eukaryotic translation initiation factor 4E-binding protein (4EBP) pathways downstream of mTORC1 to define the role of mTOR-dependent translation initiation signals in WNV gene expression and growth. We now show that WNV growth and protein expression are dependent on mTORC1 mediated-regulation of the eukaryotic translation initiation factor 4E-binding protein/eukaryotic translation initiation factor 4E-binding protein (4EBP/eIF4E) interaction and eukaryotic initiation factor 4F (eIF4F) complex formation to support viral growth and viral protein expression. We also show that the canonical signals of mTORC1 activation including ribosomal protein s6 (rpS6) and S6K phosphorylation are not required for WNV growth in these same conditions. Our data suggest that the mTORC1/4EBP/eIF4E signaling axis is activated to support the translation of the WNV genome.

## 1. Introduction

West Nile virus (WNV) is a prototypical (+) sense, single-stranded RNA virus from the family *Flaviviridae*. The viral nonstructural protein 3 contains methyltransferase [1] and 5′ RNA triphosphatase activity [2,3] to generate a 5′ capped genomic viral RNA. WNV genomic RNA possesses a canonical 7-methylguanosine (^m7^GpppN_m_)5′ cap with a 2′-*O*-methylation that mimics host mRNAs preventing innate immune detection [4] and allowing the virus to translate its RNA genome through the utilization of cap-dependent translation initiation effectors in a wide variety of hosts [5,6]. However, it is unclear whether flaviviruses are obligated to utilize specific host cap-dependent translation initiation factors in a canonical manner [7]. 

Flaviviruses do not shut down the translation of host mRNA during infection, so work to understand the mechanisms that flaviviruses manipulate to compete with host mRNA for access to the translational apparatus will provide important insight into the function of the viral RNA. We have previously shown that WNV activates mammalian target of rapamycin (mTOR) and that inhibition of mTOR results in a significant WNV growth defect associated with decreased viral protein production [8]. mTOR is a major regulator of cellular RNA translation initiation and gene expression and is highly conserved from yeast to mammals [9]. Initiation of translation is a major checkpoint of cellular control of host cell gene expression and is likely to be a major checkpoint for the control of viral gene expression. Thus, the study of viral interactions with the TOR pathway provides insight into the central features of the viral lifecycle and processes that promote the translation of viral RNA [10].

**Figure 1:**
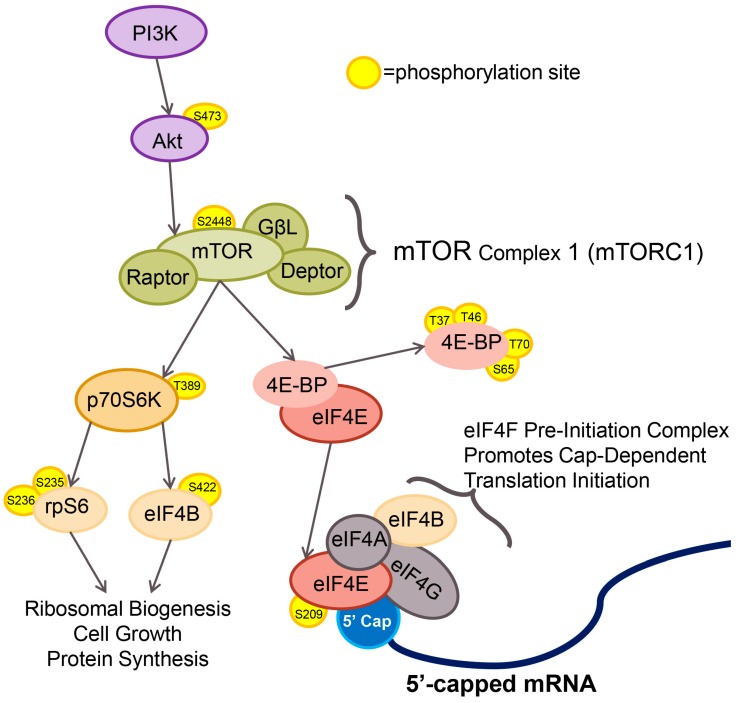
The mammalian target of rapamycin complex 1 (mTORC1) pathway and translation initiation. Cellular mTORC1 activity is regulated in part by phosphatidylinositol-3-kinase/protein kinase B (PI3K/Akt) signaling. Phosphorylation of mTOR on residue serine 2448 leads to activation of mTOR and phosphorylation of mTORC1 effector proteins, 70 kDa ribosomal protein S6 kinase 1 (p70S6K) and eukaryotic initiation factor 4E binding protein (4EBP). Phosphorylation-induced activity of p70S6K phosphorylates ribosomal protein S6 (rpS6) and eukaryotic initiation factor 4B (eIF4B) resulting in initiation of specific cap-dependent translation events. mTORC1-dependent phosphorylation of 4EBP leads to dissociation of 4EBP from eIF4E. Free eIF4E binds to the 5′ cap of host mRNAs and forms the eIF4F pre-initiation complex.

mTOR is a highly evolutionarily-conserved serine/threonine kinase that forms two distinct multi-protein complexes in higher eukaryotes: mTORC1 and mTORC2. mTORC1 includes the cofactor regulatory-associated protein of mTOR (Raptor) resulting in mTOR catalytic activity for specific targets (Figure 1). Raptor interacts with 70 kDa S6 kinase 1 (p70S6K) and the eukaryotic initiation factor 4E binding proteins (4EBP) [11,12,13,14]. 

P70S6K is a member of the S6 kinase family, which includes two constitutively-expressed genes, *S6K1* and *S6K2* [15,16]. Due to alternative splicing events, a larger protein called p85S6K1 is produced, but has an unknown function, while the smaller p70S6K protein is thought to be the functional protein for translational control [17,18]. Activation of p70S6K by mTORC1 via phosphorylation at threonine 389 promotes phosphorylation and activation of ribosomal protein S6 (rpS6) and eukaryotic initiation factor 4B (eIF4B). The phosphorylation of rpS6 enhances translation initiation by promoting the recruitment of rpS6 to the 7-methylguanosine cap complex and facilitating the assembly of the pre-initiation complex [19]. When phosphorylated, eIF4B promotes translation initiation by enhancing the helicase activity of eIF4A during scanning in the mRNA 5′ untranslated region (UTR) [20,21]. 

mTOR also regulates cap-dependent translation initiation rates through phosphorylation of the eIF4E binding proteins: 4EBP1, 4EBP2 and 4EBP3. 4EBP1 and 4EBP2 are ubiquitously expressed in higher eukaryotes and function by binding the 5′ cap binding protein eIF4E, a core component of the eukaryotic pre-initiation eIF4F complex. When hypophosphorylated, as in conditions of nutrient starvation (a known repressor of mTORC1 activity), 4EBP1 binds to eIF4E, sequesters eIF4E in the cell and limits cap-dependent translation initiation events. With the addition of nutrients, mTORC1 phosphorylates 4EBP in an ordered event, beginning with threonine 37 and threonine 46, followed by threonine 70 and serine 65 and resulting in dissociation of 4EBP from eIF4E [22,23]. The 4EBP family of proteins also regulates the activity of eIF4E by altering the cellular localization of eIF4E [24,25]. The host factor eIF4E is a key component in the eIF4F pre-initiation complex and is required for cap-dependent translation initiation (Figure 1). 

The eIF4F complex is commonly targeted by viruses that shutoff host protein synthesis to promote viral translation, but few data exist for the role of eIF4E during WNV infection [10,26,27]. 

Our previous work suggested that the mTOR-dependent activation of S6K1 and/or 4EBP play important roles in (+) strand RNA virus gene expression [8]. In this study, we used cellular gene deletion studies of the S6 kinase (S6K) and 4EBP pathways to define the role of mTOR-dependent signaling in WNV gene expression and growth. We now show that S6K/rpS6 activity does not impact WNV growth or protein expression, while loss of 4EBP function and disruption of eIF4F complex activity has a significant impact on WNV growth and protein expression. These data suggest that mTORC1/4EBP/eIF4E signaling plays an important role in modulating the intracellular environment to support WNV gene expression.

## 2. Materials and Methods 

### 2.1. Virus Propagation and Titration

West Nile virus stocks were obtained from the clone-derived 385-99 (NY99) strain and propagated in C6/36 *Aedes albopictus* (American Type Culture Collection, ATCC^®^ CRL-1660™, Manassas, VA, USA) cells as previously described [28]. Chikungunya virus (CHIKV) stocks were derived from the La Reunion 2006 OPY-1 (CHIKV-LR) strain and propagated on C6/36 cells as previously described [29]. Encephalomyocarditis virus (EMCV), strain EC9, was generated in HeLa cells (ATCC^®^, CCL-2™) transfected with pEC9 (gift from David Barton, University of Colorado, Aurora, CO, USA) to produce infectious virus stocks [30]. Syrian golden hamster kidney cells (BHK-21 [C-13]; ATCC^®^, CCL-10™) were used to measure viral titer by standard plaque assay [31]. For infection, cells were inoculated with WNV, CHIKV or EMCV and incubated at 37 °C and 5% CO_2_ for 1 h. All time points were measured from the end of the 1-h adsorption period and reported as hours post-infection (hpi).

### 2.2. Cell Lines

All cell lines were maintained at 37 °C and 5% CO_2_, except C6/36 cells, which were maintained at 28 °C. Vero (ATCC^®^ CCL-81™), C6/36 and BHK-21 cells were maintained in Dulbecco’s Modified Eagle Medium (Gibco, Grand Island, NY, USA) supplemented with 1% penicillin/streptomycin (Pen/Strep; Gibco), 10% heat-inactivated fetal bovine serum (Gibco), 1% non-essential amino acids (Gibco) and 1% sodium pyruvate (Gibco). Murine embryonic fibroblasts (MEFs, gift of Dr. Michael Hall, Freidreich Meischer Institute, Switzerland) were maintained in Dulbecco’s Modified Eagle Medium supplemented with 1/% Pen/Strep and 10% heat-inactivated fetal bovine serum.

### 2.3. Inducible Raptor Murine Embryonic Fibroblasts 

Inducible *Raptor* knockout (iRapKO) MEFs were gifts from Michael Hall of the Freidreich Meischer Institute and have been previously described in detail [32]. For induction, 2 mM 4-hydroxytamoxifen (Sigma-Aldrich, St. Louis, MO, USA) suspended in 95% ethanol was diluted in MEM to a final concentration of 1 μM. At 24 h post-induction, cells were treated with trypsin (Gibco), counted and plated at 80,000 cells/well in a 6-well plate format or 5000 cells/well in an 8-well chamber slide format. Cells were allowed 48 additional hours before experimental manipulation to allow for full induction of *Raptor* gene knockout. Cells were infected with 1 × 10^6^ plaque-forming units (pfu) of WNV/CHIKV/EMCV per well (6-well plate format, multiplicity of infection (MOI) = 3) or 5 × 10^4^ pfu WNV/well (8-well chamber slide format, MOI = 3) for 1 h at 37 °C and 5% CO_2_.

### 2.4. Western Blots

Cells were harvested by dissociating with trypsin at indicated times post-infection, suspended in ice-cold 1X phosphate buffered saline (PBS; Gibco) centrifuged at 3000× *g* for 5 min, and pellets suspended in lysis buffer (Cell Signaling Technology, Boston, MA, USA), plus Halt protease and phosphatase inhibitor cocktail (Thermo Scientific, Rockford, IL, USA) and disrupted using an ultrasonic processor VCX130 (Sonics & Materials, Newtown, CT, USA) for one cycle × 10 s. Whole cell extracts were run on standard 10% sodium dodecyl sulfate-polyacrylamide gel electrophoresis (SDS-PAGE) gels (Criterion system; Bio-Rad, Hercules, CA, USA). The separated proteins were electrically transferred to polyvinylidene fluoride 0.45-µm pore membranes (Millipore, Billerica, MA, USA) at 100V for 1 h. For all Western blot (WB) analyses, membranes were activated for 10–15 s in methanol, blocked for 1 h with 5% Blocking Grade Buffer (Bio-Rad) post-transfer, and probed with primary antibodies: rabbit monoclonal antibody to ribosomal protein S6, Raptor, 4EBP1, phospho-4EBP1 (T37/46), mTOR and β-actin; rabbit polyclonal to phospho-p70 S6 kinase (T389), phospho-ribosomal protein S6 (S235/236), p70 S6 kinase, phospho-eIF4B (S422) and eIF4B (Cell Signaling Technology). Additional polyclonal rabbit WNV NS3 antibody was a generous gift from Aaron Brault (Centers for Disease Control (CDC), Ft. Collins, CO, USA). For CHIKV antibody, we obtained mouse immunoglobulin from ascites to CHIKV antigen (V-548-701-562; ATCC^®^ VR-1241AF™). After washing, membranes were probed with appropriate horseradish peroxidase (HRP)-conjugated secondary antibodies (Jackson ImmunoResearch, West Grove, PA, USA) and images obtained after incubation with Western Lightning ECL Pro (Perkin Elmer, Waltham, MA, USA) and visualized with the ChemiDoc XRS+ system (Bio-Rad). Band density was calculated using ImageJ analysis (version 1.47, Rasband, W.S., ImageJ, U. S. National Institutes of Health, Bethesda, Maryland, USA, http://imagej.nih.gov/ij/, 1997-2016) and corrected for β-actin band density to provide mean band density for each Western blot replicate.

### 2.5. RNA Isolation and qRT-PCR

Cells were harvested at the indicated time post-infection and RNA isolated using the RNeasy Mini Kit (Qiagen, Hilden, Germany) per the manufacturer’s instructions and stored at −80 °C until cDNA library generation. cDNA libraries were generated from total RNA isolates using the Super Script III First-Strand Synthesis System (Life Technologies, Carlsbad, CA, USA) as per the manufacturer’s protocol using random hexamer primers. CHIKV quantitative reverse-transcription polymerase chain reaction (qRT-PCR) was accomplished using previously described methods [33].

### 2.6. MTT Assay

MTT (3-(4,5-dimethylthiazol-2-yl)-2,5-diphenyltetrazolium bromide) assays were conducted as per the manufacturers’ protocols (Sigma-Aldrich, Cat. No. M5655). In brief, the MTT assay was conducted in 6-well plates, formazan crystal solubilized in acidified isopropanol and read in 96-well plates using the VictorX5 plate reader (Perkin Elmer). Uninfected, non-treated Vero cells were utilized as a viable-cell control, and a cell-free media control was included to correct for background absorbance.

### 2.7. Biochemical Inhibitor Studies

The eIF4F complex-formation inhibitor 4EGI-1 was obtained from Tocris Biosciences (Avonmouth, Bristol, UK) (CAS No. 315706-13-9/0). The compound was suspended in dimethyl sulfoxide (DMSO; Sigma) to make a 1000X stock at a concentration of 100 mM and diluted to final concentrations in DMEM (Gibco). Fresh solutions of the compound were prepared for individual experiments. A DMSO vehicle control was included in all studies.

### 2.8. Statistical Analysis

All data were analyzed using Prism software (GraphPad Prism6, La Jolla, CA, USA) using the indicated statistical analysis tests. Statistical significance was determined with an alpha less than 0.05. 

## 3. Results

### 3.1. Raptor Deletion Reduces the Growth of 5′-Capped Viruses, but Not an IRES-Translated Virus

We previously demonstrated that WNV activates mTORC1 activity in several cell types and that mTORC1 activity supports viral growth and protein expression [8]. To investigate the role of cap-dependent signals in viral protein expression, we used an iRapKO MEF model that was previously described [32,34]. By knocking out the cofactor Raptor, we were able to specifically eliminate mTORC1 activity with no effect on mTOR or mTORC2 activity. Using the iRapKO system, we determined the effect of mTORC1 activity on multiple rounds of WNV replication using a low multiplicity of infection. Next, we determined whether the effect of Raptor deletion on viral growth and protein expression was specific for just WNV or played a role in pathogenesis for other capped RNA viruses. Thus, we used CHIKV (a 5′-capped RNA virus) and EMCV as additional controls. CHIKV utilizes a cap-dependent translation system for the translation of nonstructural proteins and uses a capped-subgenomic mRNA to express structural proteins. EMCV initiates translation using an internal ribosome entry site (IRES). 

**Figure 2:**
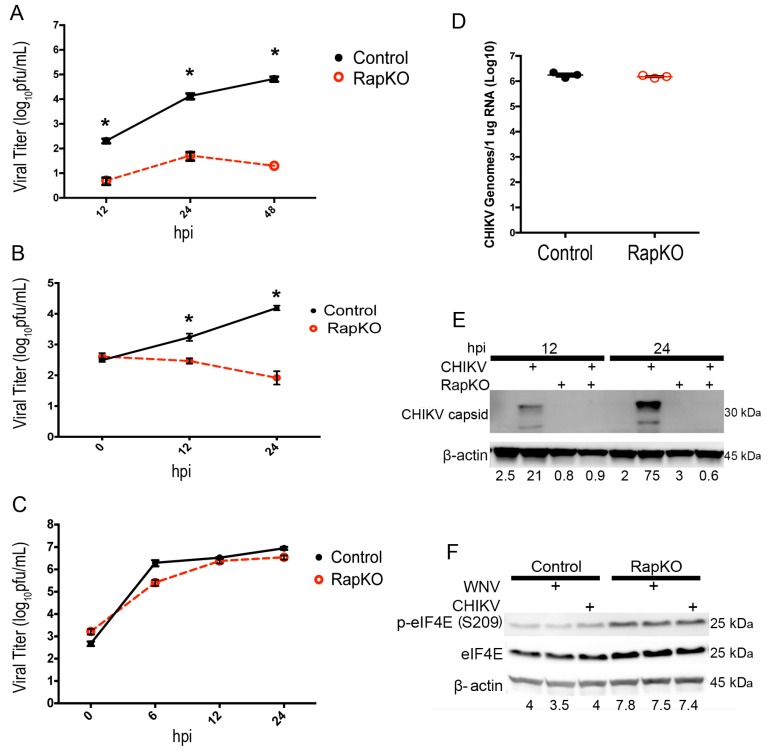
Raptor expression supports the growth and protein expression of 5′-capped RNA viruses. (**A**) Multi-step growth curve (MOI = 0.001) of West Nile virus (WNV) in control and Raptor knockout (RapKO) cells. *N* = 6 replicates per time point, * *p* < 0.0001; a single-step growth curve (MOI = 3) is shown for (**B**) Chikungunya virus La Reunion 2006 OPY-1 (CHIKV-LR) and (**C**) encephalomyocarditis virus (EMCV) in control and RapKO cells as determined by standard plaque assay of the supernatant. The viral titer is presented as log10 pfu/mL at indicated times (hours post-infection (hpi)). *N* = 6 replicates per time point, * *p* < 0.005; (**D**) Cell-associated CHIKV-LR genome copies in RapKO and control murine embryonic fibroblast (MEF) cells at 0 hpi determined by quantitative reverse-transcriptoin PCR (qRT-PCR). Data are presented as log10 CHIKV-LR genomes/µg of RNA; (**E**) Western blot (WB) analysis of CHIKV-LR protein synthesis in RapKO cells. Cellular lysates were harvested at the indicated time (hpi), normalized for total protein and subjected to WB analysis using antibodies to CHIKV capsid protein and a β-actin loading control. Images are representative of two independent experiments. Densitometry values are provided for CHIKV capsid corrected for β-actin expression; (**F**) Expression of phospho-eIF4E (serine 209), total eIF4E and β-actin in control and RapKO cells as determined by Western blot analysis. Total protein lysate was collected from cells either mock, WNV or CHIKV-LR infected (MOI = 3), separated by gel electrophoresis and probed for the indicated antibody targets. Images are representative of two independent experiments. Densitometry values are provided for p-eIF4E expression corrected for β-actin expression.

First, we completed a multi-step growth curve with WNV infection to evaluate the role of Raptor expression during multiple WNV replication cycles. iRapKO cells were treated with 4-hydroxytamoxifen (4-OHT) to induce knockout or mock-induced with ethanol as a control. Cells were then inoculated with WNV (MOI 0.001) at 72 h post-induction, and the virus-containing supernatant was collected and titered. WNV growth was significantly reduced in iRapKO cells at 12 h (5 pfu/mL ± 1.6 (mean ± standard error of the mean (SEM)), 24 h (52.3 pfu/mL ± 19.8) and 48 h (20 pfu/mL ± 5.2) post-infection, compared to the ethanol-treated control cells at 12 h (205.3 pfu/mL ± 42.8), 24 h (1.4 × 10^4^ pfu/mL ± 3619) and 48 h (6.7 × 10^4^ pfu/mL ± 1.5 × 10^4^) post-infection (*p* < 0.0001, *n* = 6 per group, two-way ANOVA; Figure 2A). 

Next, we evaluated the role of Raptor expression on CHIKV growth to define the role of mTORC1 on a different positive-strand RNA virus. iRapKO MEFs were treated with the inducing agent 4-OHT or ethanol vehicle control as above, were inoculated 72 h post-induction with CHIKV-LR (MOI = 3), and the supernatant was collected for the standard viral titer determination. In iRapKO cells, CHIKV-LR growth was significantly (*p* = 0.005) inhibited at 12 h (3 × 10^2^ pfu/mL (mean) ± 1.23 (SEM)) and 24 h (*p* < 0.0001, 8 × 10 pfu/mL ± 1.6) post-infection compared to ethanol-treated, control cells at 12 h (1.6 × 10^3^ pfu/mL ± 16) and 24 h post-infection (1.6 × 10^4^ pfu/mL ± 1.2, *n* = 3, two-way ANOVA; Figure 2B). In CHIKV-LR-inoculated, iRapKO cells, the virus did not show evidence of growth following the t = 0 h titer of 4 × 10^2^ pfu/mL. 

Since CHIKV-LR did not exhibit evidence of viral growth in iRapKO cells, we next evaluated iRapKO cells for the ability to support viral growth of an IRES-driven EMCV isolate. Using the same treatment as described above, we inoculated control and iRapKO MEF cells with EMCV (MOI = 3) and harvested the supernatant at specific time points post-infection. We found no evidence of a growth defect in EMCV-inoculated iRapKO cells at 6 h (2.6 × 10^5^ pfu/mL ± 8.1 × 10^4^), 12 h (2.6 × 10^6^ pfu/mL ± 5.5 × 10^8^) or 24 h (3.5 × 10^6^ pfu/mL ± 7.5 × 10^5^) post-infection when compared to EMCV-inoculated ethanol-control treated MEF cells at 6 h (2 × 10^6^ pfu/mL ± 6 × 10^5^), 12 h (3.3 × 10^6^ pfu/mL ± 2.4 × 10^5^) and 24 h (8.9 × 10^6^ pfu/mL ± 1.3 × 10^6^) post-infection (*p* = 0.5988, *n* = 3, two-way ANOVA; Figure 2C). These data demonstrated that EMCV, a virus that depends on IRES-initiated translation, exhibits no significant growth defect when mTORC1 signaling is disrupted through Raptor knockout.

To further evaluate the CHIKV-LR growth defect in iRapKO cells, we determined if iRapKO cells were infected at the same level as control cells. iRapKO MEFs were treated with 4-OHT or ethanol vehicle control as above, and then inoculated 72 h post-induction with CHIKV (MOI = 3); cells were washed twice with PBS after one hour absorption as above, and harvested at t = 0 post-infection to evaluate CHIKV RNA using quantitative-reverse transcriptase PCR. We found a mean of 1.3 × 10^6^ copies of CHIKV RNA/1 µg of RNA in both iRapKO cells and vehicle control cells (Figure 2D). This experiment was repeated in triplicate using a low pH wash instead of PBS to remove bound virus that had not entered the cell. We found no difference in the entry of CHIKV when comparing CHIKV copies/µL at t = 0 post-infection in iRapKO cells (3.5 × 10^4^ (mean)) to vehicle control cells (3.42 × 10^4^).

While CHIKV-LR-inoculated iRapKO cells were RNA-positive, the virus seemed unable to replicate in these cells. To determine if this was related to an effect on CHIKV protein production, we inoculated the same treatment groups described above with CHIKV-LR (MOI = 3) and harvested cells at 12 h and 24 h post-infection for total protein analysis. Western blot analysis of whole cell lysates revealed that CHIKV-inoculated, iRapKO cells were not expressing CHIKV capsid protein following infection (Figure 2). However, control cells expressed high levels of CHIKV capsid protein. These data suggest that targeted Raptor gene deletion to eliminate mTORC1 activity in MEF cells diminished CHIKV protein production.

Previous reports suggest differing roles of mTORC1 activity in CHIKV growth and eIF4E phosphorylation, so we examined the phosphorylation status of eIF4E in iRapKO MEFs [35,36]. Inhibition of mTORC1 may enhance eIF4E phosphorylation at serine 209 in certain cell types and may play a role in viral RNA translation [35,37]. To assess whether eIF4E phosphorylation occurred under our experimental conditions, we harvested cellular lysates from control and iRapKO MEFs infected with CHIKV-LR and WNV (MOI = 3) at 12 hpi. We found that eIF4E phosphorylation occurs in iRapKO conditions at 12 hpi as previously reported and is independent of infection status [35]; but phosphorylation of eIF4E in iRapKO cells did not compensate for the lack of mTORC1 signaling to promote CHIKV protein production in our studies (Figure 2F). 

### 3.2. Impact of Raptor Knockout on mTORC1 Effectors

**Figure 3:**
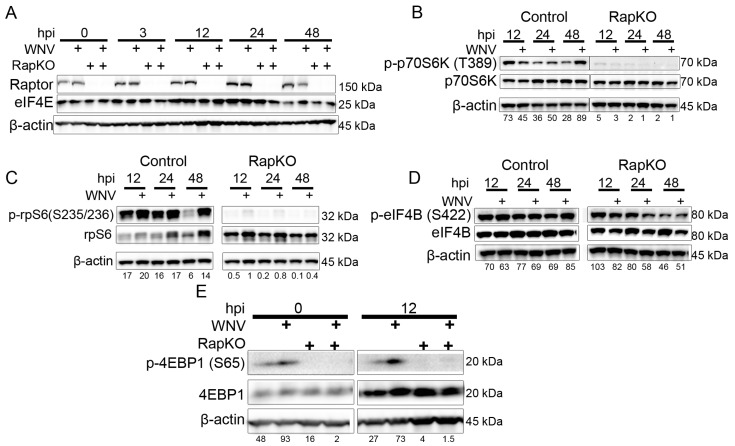
Raptor knockout results in a loss of p70S6K and 4EBP phosphorylation. Cells were either mock- or WNV-inoculated (MOI = 3) in control and iRapKO MEF cells, total cellular protein lysates were collected at the indicated hpi, and probed using antibodies for the indicated target proteins by western blot analysis. (**A**) Expression of Raptor and eIF4E; (**B**) Expression of phospho-p70S6K (tyrosine 389) and total p70S6K; (**C**) Expression of phospho-rpS6 (serine 235/236) and total rpS6. (**D**) Expression of phospho-eIF4B (serine 422) and total eIF4B; (**E**) Expression of phospho-4EBP1 (serine 65) and total 4EBP1. All images are representative of two independent experiments. β-actin was used as the loading control for all experiments. Densitometry values are provided for phospho-protein expression and corrected for β-actin expression.

Our data show that mTORC1 has a significant impact on viral growth and viral protein production. We next defined the role of the primary mTORC1 downstream targets, S6K and 4EBP, in viral protein production. We inoculated ethanol-induced control cells and iRapKO cells as above with mock or WNV (MOI = 3) inoculums and harvested whole cell lysates at 0, 3, 12, 24 and 48 h post-infection. Western blot analysis for Raptor protein expression and eIF4E expression revealed that knockdown of Raptor expression in iRapKO cells had no significant impact on total eIF4E expression in WNV-infected cells compared to mock-inoculated cells (Figure 3A).

We previously reported that WNV infection induced p70S6K activation in serum-starved cells [8]. We extended these data by determining the role of downstream effectors of mTORC1 activity. Control and iRapKO cells were inoculated with mock or WNV (MOI = 3) as above, and cells were harvested for Western blot analysis at 12, 24 and 48 h post-infection (Figure 3B–D). On Western blot analysis, control cells exhibit phosphorylation of mTORC1 effector p70S6K at T389, while iRapKO cells exhibit loss of p-p70S6K expression, but unchanged total p70S6K expression in both the mock- and WNV-infected groups (Figure 3B). Thus, infection with WNV does not stimulate phosphorylation of p70S6K via an alternative pathway. 

Following WNV infection of control MEF cells, total rpS6 expression was increased at 24 and 48 h post-infection, while phosphorylated rpS6 at S235/236 was increased at 48 h post-infection when compared to mock-infected cells (Figure 3C). In WNV-inoculated iRapKO cells, phosphorylated rpS6 expression was decreased at all time points, and total rpS6 expression increased across all time points and treatments (Figure 3C). While S6K and rpS6 are exclusively phosphorylated by mTORC1, eIF4B is phosphorylated by mTORC1 and other cellular kinases. Thus, we evaluated the phosphorylation status of eIF4B at serine 422 in the previously-described treatment groups and time points. We found that total and phosphorylated eIF4B expression was not significantly altered by WNV infection or Raptor expression (Figure 3D). These data show that iRapKO has targeted effects on the expression of downstream S6K-dependent signaling events with little off-target effects. 

We next defined the role of Raptor expression and WNV infection on 4EBP expression, the other primary target of mTORC1 activity. Control and iRapKO cells were inoculated with mock or WNV (MOI = 3) and whole cell lysates harvested at 0 h and 12 h post-infection (Figure 3E). Western blot analysis revealed loss of 4EBP phosphorylation signal at 0 h and 12 h post-infection in WNV-inoculated iRapKO cells, while total 4EBP levels remained stable between the two conditions (Figure 3E). In WNV-inoculated control cells, we also found evidence of increased phosphorylation of 4EBP at serine 65. Since this is the terminal phosphorylation event in sequential 4EBP phosphorylation, these data suggest that WNV-induced mTORC1 activity results in increased 4EBP phosphorylation with a subsequent increase in unbound eIF4E, cap-binding protein. Overall, these data show that loss of mTORC1 activity in this system results in decreased signaling events through the S6K/rpS6 and 4EBP pathways in iRapKO cells. 

### 3.3. Loss of S6K/rpS6 Activity Does Not Impact WNV Growth

**Figure 4:**
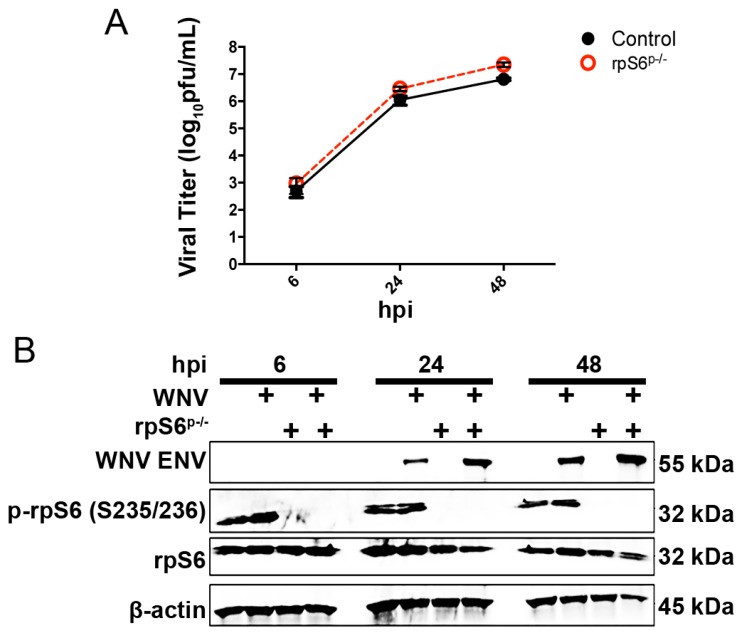
Loss of rpS6 phosphorylation does not impact WNV growth or protein expression. (**A**) Single-step growth curve of WNV (MOI = 3) in rpS6^p−/−^ and wild-type control MEFs as determined by standard plaque assay of the cell supernatant; (**B**) expression of the WNV envelope (WNV ENV), phospho-rpS6 (serine 235/236) and total rpS6 as determined by Western blot analysis of mock- and WNV-infected (MOI = 3) rpS6^p−/−^ and wild-type cells. Total cellular protein lysates were collected at the indicated hpi and probed using antibodies against the indicated targets. β-actin was used as the loading control. Images are representative of two independent experiments.

Next, we defined the role of the mTORC1/S6K/rpS6 signaling pathway in WNV protein expression. We first determined the role of rpS6 phosphorylation at serine 235 and serine 236 by using a MEF cell line that expresses an alanine substitution at positions 235 and 236 in the rpS6 (rpS6^p−/−^) protein [38]. RpS6^p−/−^ and matched, wild-type MEFs (rpS6^p+/+^) were infected with mock or WNV (MOI = 3); supernatants and whole cell lysates were collected for viral titer analysis and Western blot at 6, 24 and 48 h post-infection. Viral titer analysis revealed no significant difference in WNV growth over a 48-h period (*p* = 0.30, two-way ANOVA, *n* = 11; Figure 4A). Western blot analysis of whole cell lysates at the same time points revealed no significant change in WNV envelope (ENV) protein expression despite loss of phosphorylation of rpS6 at both residues 235 and 236 (Figure 4B). 

Although rpS6 is involved in the initiation complex, these results indicated that rpS6 phosphorylation is dispensable for the support of WNV protein expression [19].

**Figure 5:**
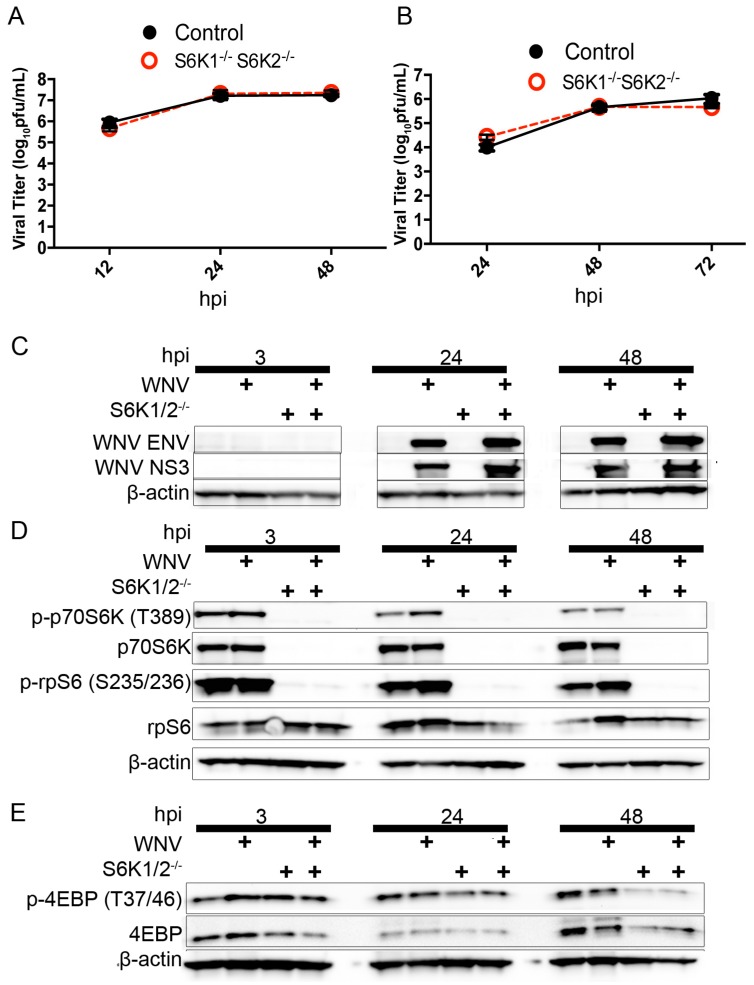
P70S6K activity does not support WNV growth or protein expression. (**A**) Single-step growth curve of WNV (MOI = 3) in S6K1/2^−/−^ and wild-type control MEFs; (**B**) Multi-step growth curve (MOI = 0.001) of WNV in S6K1/2^−/−^ and wild-type control MEFs. Supernatants were harvested at the indicated hpi for standard plaque assay. *N* = 6 replicates per time point; (**C**) WB analysis of mock- and WNV-infected (MOI = 3) S6K1/2^−/−^ and wild-type cells for WNV protein expression. Cellular lysates were collected at the indicated hpi and probed using antibodies for WNV ENV, WNV NS3, and β-actin loading control. Representative of 2 independent experiments; (**D**) WB analysis of mock- and WNV-infected (MOI = 3) S6K1/2^−/−^ and wild-type cells for rpS6 and p70S6K expression and phosphorylation. Cellular lysates were collected at the indicated hpi and probed using antibodies against p-p70S6K [T389], total p70S6K, p-rpS6 [S235/236], total rpS6, and β-actin loading control. Representative of two independent experiments; (**E**) WB analysis of mock- and WNV-infected (MOI = 3) S6K1/2^−/−^ and wild-type cells for 4EBP expression and phosphorylation. Cellular lysates were collected at the indicated hpi and probed using antibodies for p-4EBP [T37/46], total 4EBP, and β-actin loading control. Representative of two independent experiments.

Next, we obtained S6K1/2^−/−^ MEFs as previously described to determine the contribution of S6K1 and S6K2 signaling to WNV protein expression [39]. We used an S6K1 and S6K2 double knockout MEF cell line (S6K1/2^−/−^) because S6K1 and S6K2 are capable of compensatory upregulation [16]. Syngeneic control MEF cells and S6K1/2^−/−^ MEFs were inoculated with WNV (MOI = 3) and supernatants obtained at 12, 24 and 48 h post-infection for viral titer analysis. We found no significant difference in WNV growth comparing S6K1/2^−/−^ and S6K1/2^+/+^ control MEF cells (*p* = 0.17, two-way ANOVA, *n* = 6, Figure 5A). 

Next, we evaluated the role of S6K expression in WNV growth following multiple steps of virus entry, replication, and egress using a multi-step growth curve. Syngeneic control MEF cells and S6K1/2^−/−^ MEFs were inoculated with WNV (MOI = 0.001) and supernatants obtained at 24, 48 and 72 h post-infection for viral titer analysis. We found no significant difference in WNV growth comparing S6K1/2^−/−^ and S6K1/2^+/+^ control MEF cells (*p* = 0.2, two-way ANOVA, *n* = 6, Figure 5B). Next, we inoculated S6K1/2^−/−^ and S6K1/2^+/+^ MEF cells with mock or WNV (MOI = 3) and harvested whole cell lysates for Western blot analysis at 3, 24 and 48 h post-infection. We analyzed the expression of both nonstructural protein 3 (NS3) and ENV proteins from WNV. Both proteins are translated from the same polyprotein, but the individual fate of these proteins is different, with NS3 remaining intracellular; and ENV structural protein is packaged during viral egress. This approach allows for an additional control by comparing ENV, a protein exported out of the cell, to NS3, a protein that accumulates within the cell. We found that control and S6K1/2^−/−^ MEFs exhibited similar levels of WNV NS3 and ENV protein expression at 24 and 48 h post-infection (Figure 5C). We also completed Western blot analysis for S6K and 4EBP signaling events in mock- and WNV-infected (MOI = 3) control and S6K1/2^−/−^ MEFs at 3, 24 and 48 h post-infection. We found that knockout of S6K1 and S6K2 eliminated the expression of phosphorylated p70S6K, total p70S6K and phosphorylated rpS6 at all time points and in all treatment groups (Figure 5D). Total rpS6 expression was unchanged by S6K1/2 knockout. In the absence of S6K protein expression, we also determined if 4EBP phosphorylation was increased due to decreased competition for mTORC1-dependent phosphorylation of 4EBP from S6K. In the same treatment groups, we found no evidence of increased 4EBP phosphorylation in the S6K1/2^−/−^ MEFs compared to controls (Figure 5E). 

### 3.4. Knockout of 4EBP Expression Leads to Decreased WNV Growth and Decreased Viral Protein Production

**Figure 6:**
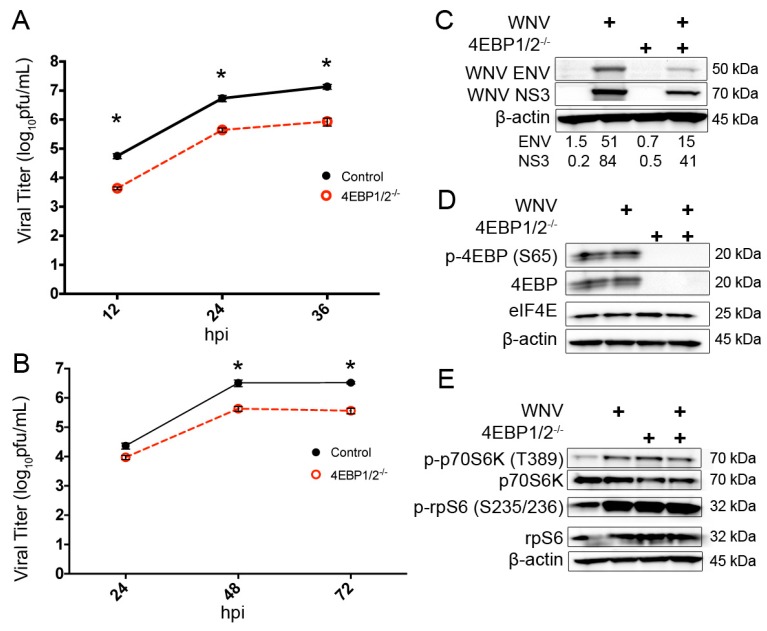
Expression of 4EBP1 and 4EBP2 support WNV growth and protein expression. (**A**) Single-step growth curve of WNV (MOI = 3) in 4EBP1/2^−/−^ and wild-type control MEFs; (**B**) Multi-step growth curve of WNV (MOI = 0.001) in 4EBP1/2^−/−^ and wild-type control MEFs. Supernatants were harvested at indicated hpi for standard plaque assay analysis. *n* = 6, * *p* < 0.0001; (**C**) Confirmation of 4EBP1/2^−/−^ (knockout) phenotype. Cellular lysates were harvested at 24 hpi and WB analysis completed using antibodies for total 4EBP, phospho-4EBP (serine 65) and eIF4E. (**D**) WNV protein synthesis in 4EBP1/2^−/−^ and matched control MEF cells. Cellular lysates were harvested at 24 hpi and subjected to WB analysis using antibodies against WNV NS3 and WNV ENV. (**E**) S6K pathway activity in 4EBP1/2^−/−^ and matched control MEF cells. Cellular lysates were harvested at 24 hpi for WB analysis using antibodies against total p70S6K, p-p70S6K (threonine 389), total rpS6, and p-rpS6 (serine 235/236). All images representative of two independent experiments. β-actin is shown as a loading control for all western blots. Densitometry values provided for indicated viral proteins corrected for β-actin expression.

We have shown that mTORC1 activity supports the expression of WNV proteins; however, loss of the mTORC1 effectors S6K and phosphorylated rpS6, both of which regulate translation initiation rates, had no significant impact on WNV growth or protein expression. Next, we inoculated 4EBP1/2^−/−^ MEF cells and matched 4EBP1/2^+/+^ control cells with WNV (MOI = 3) and collected the supernatant for viral titer analysis. Input virus was assayed using reverse transcription PCR (RT-PCR) and found to be equivalent at t = 0 (data not shown). WNV growth was significantly (*p* < 0.0001) decreased in WNV-infected 4EBP1/2^−/−^ MEF cells at 12 h (4.3 × 10^3^ pfu/mL ± 3.8 × 10^2^), 24 h (4.4 × 10^5^ pfu/mL ± 7 × 10^4^), 36 h (8.6 × 10^5^ pfu/mL ± 2 × 10^5^) and 48 h (2.2 × 10^6^ pfu/mL ± 1.7 × 10^5^) compared to control cells at 12 h (5.2 × 10^4^ pfu/mL ± 9.6 × 10^3^), 24 h (5.4 × 10^6^ pfu/mL ± 1.2 × 10^6^), 36 h (1.4 × 10^7^ pfu/mL ± 2.5 × 10^6^) and 48 h (1.8 × 10^7^ pfu/mL ± 2.5 × 10^6^, *n* = 6, two-way ANOVA; Figure 6A). 

Thus, WNV-infected 4EBP1/2^−/−^ MEF cells exhibit an 8–16-fold reduction in viral growth at all time points compared to wild-type control MEF cells. To evaluate the effect of 4EBP expression on WNV growth over multiple cycles of viral entry, replication and egress, we used a multi-step growth curve analysis. We inoculated 4EBP1/2^−/−^ MEF cells and matched 4EBP1/2^+/+^ control cells with WNV (MOI = 0.001) and collected supernatant for viral titer analysis. We found that WNV growth was significantly (*p* < 0.0001) decreased at the 48-h and 72-h time points of infection with a mean difference of 2.8 × 10^6^ pfu/mL ± 5 × 10^5^ and 2.9 × 10^6^ pfu/mL ± 5 × 10^6^, respectively (two-way ANOVA, *n* = 6; Figure 6B). 

Next, we inoculated 4EBP1/2^−/−^ and control MEFs with WNV (MOI = 3) or mock treatments as above and harvested cellular lysates at 24 h post-infection for Western blot analysis. We found that 4EBP1/2^−/−^ MEF cells exhibit loss of terminal phosphorylation (serine 65) of 4EBP and loss of total 4EBP expression with no change in the expression of eIF4E (Figure 6C). Interestingly, these data imply that 4EBP binding has no effect on the turnover of total eIF4E. Despite ongoing expression of eIF4E, we found significantly decreased expression of the WNV envelope protein and to a lesser extent NS3 in 4EBP knockout cells compared to control cells (Figure 6D). Despite significant changes to 4EBP signaling, 4EBP1/2^−/−^ MEFs exhibit no significant alterations in the expression of phosphorylated p70S6K at threonine 389, total p70S6K, phosphorylated rpS6 at serine 235 and 236 and total rpS6 (Figure 6E). These data suggest that mTORC1-dependent interactions with 4EBP/eIF4E support WNV growth in host cells.

### 3.5. eIF4F Complex Formation Supports WNV Virus Growth and Protein Expression

Since 4EBP is a major regulator of initiation complex (eIF4F) formation, we determined the role of eIF4F complex formation on WNV growth and protein expression by using the inhibitor 4EGI-1. 4EGI-1 blocks the interaction between eIF4E and eIF4G, thereby preventing the formation of the pre-initiation complex on mRNA [40]. This compound is a valuable tool for defining how viruses interact with eIF4E and the eIF4F complex to facilitate translation [41]. 

**Figure 7:**
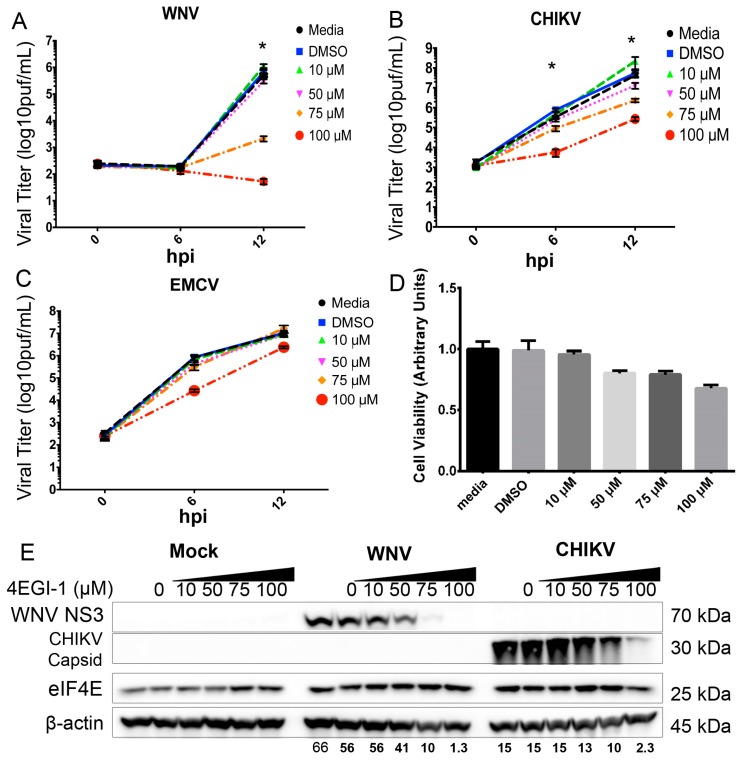
eIF4F complex formation supports 5′-capped positive-strand RNA viral growth and protein expression. Single-step growth curves (MOI = 3) for (**A**) WNV, (**B**) CHIKV-LR and (**C**) EMCV in 4EGI-treated Vero cells as determined by standard plaque assay of supernatants. 4EGI-1 was added at the indicated micromolar (µM) concentrations at t = 0 hpi, and supernatants were analyzed by plaque assay at the indicated times. Viral titer data are presented as log10 plaque forming units per mL of supernatant (log10 pfu/mL). *N* = 9–12 replicates per time point, * *p* < 0.05; (**D**) MTT assay for cellular viability of 4EGI-treated Vero cells. MTT cleavage products were solubilized and measured by spectrophotometry at 570 nm. Dimethyl sulfoxide (DMSO) was used as a solvent control, and all groups were normalized to a media control of 1.0 arbitrary units; (**E**) Western blot analysis of viral protein synthesis in mock, WNV-infected (MOI = 3) and CHIKV-infected (MOI = 3) Vero cells treated with the 4EGI-1 inhibitor. Vehicle control denoted by 4EGI-1 concentration of 0 µM. Images are representative of two independent experiments. Densitometry values provided for indicated viral proteins corrected for β-actin expression.

Vero cells were inoculated with WNV, CHIKV-LR or EMCV at an MOI of three, and viruses were allowed to adsorb on the monolayer for 1 h at 37 °C. After incubation, cells were washed with warmed 1× PBS and media containing 4EGI-1 at concentrations of 100, 75, 50 and 10 µM, a DMSO vehicle control (0 µM) or an untreated control infection. Supernatants were harvested at 0, 6 and 12 hpi and analyzed to determine viral titer. We found that treatment with 4EGI-1, in a dose-dependent manner, significantly (*p* < 0.0001) decreased WNV growth at 12 h with 4EGI-1 concentrations of 75 µm (2.1 × 10^3^ pfu/mL ± 482) compared to untreated controls at 12 h treated with PBS (5.1 × 10^5^ pfu/mL ± 6.7 × 10^4^) and DMSO (6.7 × 10^5^ pfu/mL ± 1.3 × 10^5^, *n* = 12, two-way ANOVA with multiple comparisons; Figure 7A).

Thus, there was a 242-fold decrease in WNV growth in the 75 µm 4EGI-1 treatment group. As described above, we used CHIKV infection as an additional control for capped-RNA virus growth to ensure that the broad effect of 4EGI-1 was not specific to WNV. We found that treatment with 4EGI-1, in a dose-dependent manner, significantly (*p* < 0.05) decreased CHIKV-LR growth at 6 h and 12 h with a 4EGI-1 concentration of 75 µm (2.3 × 10^6^ pfu/mL ± 4.7 × 10^5^) compared to controls at 12 hpi that were treated with PBS (4.3 × 10^7^ pfu/mL ± 1.1 × 10^7^) and DMSO (5.4 × 10^7^ pfu/mL ± 1 × 10^7^, *n* = 9, two-way ANOVA with multiple comparisons; Figure 7B). Next, we utilized EMCV infection as a control for toxicity and cap-dependent translation since EMCV protein production is regulated by IRES-dependent initiation and 4EGI-1 should have no effect on EMCV growth. We found that EMCV growth was independent of 4EGI-1 treatment and exhibited no significant (*p* > 0.05) decrease in viral growth between treatment groups (*n* = 12, two-way ANOVA; Figure 7C). Previous work has suggested that EMCV growth is independent of eIF4F complex formation [42]. Since the 6-h time point appeared to exhibit decreased EMCV growth at the 100-µm dose of 4EGI-1, we completed a multiple comparisons test of our two-way ANOVA and found no significant difference between the vehicle-treated control and the 100-µm dosed 4EGI-1 inhibitor despite 12 replicate experiments. However, the decrease in EMCV growth at this time point may be related to toxicity at the higher dose of 4EGI-1. Thus, we completed toxicity assays for the 4EGI-1 inhibitor.

To evaluate cellular viability following 4EGI-1 treatments, we completed MTT assays at 12 h post-treatment with indicated doses of inhibitor. As shown in Figure 7D, 4EGI-1 had no statistically-significant impact on cellular viability at concentrations below 100 µM in our Vero cell system. Cells treated with the highest levels of 4EGI-1 (100 µM) exhibited 65% viability compared to untreated controls at 12 h post-treatment. Based on this toxicity data, we analyzed only 4EGI-1 doses below the 100 µM dose for viral growth studies. These data show that following 4EGI-1 treatments below the 100-µm dose, cells were viable and capable of biosynthetic processes. This conclusion is supported by evidence of EMCV growth following 4EGI-1 treatment at doses of inhibitor below 100 µM.

Next, we determined the effect of 4EGI-1 treatment on WNV and CHIKV protein production. Cells were inoculated with mock inoculum, WNV (MOI = 3) or CHIVK (MOI = 3) and incubated for 1 h as above. After washing cells, medium was added with the indicated concentrations of 4EGI-1 inhibitor (Figure 7E). Cells were harvested at 12 h post-infection and lysates processed for Western blot analysis using antibodies to WNV NS3 protein, CHIKV capsid and total eIF4E. We found that treatment with 4EGI-1 results in a dose-responsive loss of viral protein production for both WNV- and CHIKV-inoculated cells (Figure 7E). This effect was independent of the effects on total eIF4E production indicating that the 4EGI-1 inhibitor disrupted initiation complex formation without altering the expression of cap-binding proteins like eIF4E. These data are in agreement with viral growth data that show 4EGI-1 dose-responsive inhibition of CHIKV and WNV growth.

## 4. Discussion

Flaviviruses have limited genomic coding capacity and, thus, have evolved mechanisms designed to utilize the host RNA translation machinery to successfully express viral proteins. The mechanisms by which (+) sense, capped RNA viruses recruit translation initiation factors to the viral RNA to promote translation has remained incompletely defined. Moreover, arboviruses, such as CHIKV and WNV, must translate their genomes in evolutionally-distant vertebrate and invertebrate hosts and, therefore, must be able to manipulate the translational apparatus in very disparate systems [6]. We now show that WNV growth and protein expression is dependent on specific host-cell initiation events that involve interactions with the 4EBP and the eIF4F complex. We think these interactions are specific for the virus because other cellular translation events downstream of S6 kinase are not required for WNV gene expression. These data show that WNV utilizes specific translation initiation signals to support viral protein production. From an evolutionary standpoint, some RNA viruses, like the arboviruses, may modulate the TORC1/4EBP/eIF4E pathway because this system regulates cellular cap-binding events and is highly conserved in eukaryotic cells from yeast to mammals [9,43]. 

Previously published results have demonstrated that WNV infection induces the activation of the mTORC1/S6K signaling pathway as early as 3 h post-infection, but the cap-dependent translation initiation effectors downstream of TORC1 responsible for viral growth were not known [8]. In this study, we demonstrate that WNV depends on mTORC1/Raptor activity for viral growth over multiple life cycles of the virus. This effect may apply broadly to 5′-capped-RNA viruses, such as CHIKV, since we found that CHIKV is unable to sustain growth in the absence of mTORC1 activity. However, we show that viruses like EMCV that initiate viral RNA translation using an IRES replicate in the absence of TORC1 activity. Recent work studying the interactions between CHIKV and mTOR signaling have revealed differing conclusions for the role of mTORC1 activity in CHIKV growth [35,36]. Our studies build upon this existing body of work and demonstrate that capped RNA viruses, such as WNV and CHIKV are dependent on mTORC1 signaling to support viral growth independent of eIF4E phosphorylation status. We found evidence of phosphorylation of eIF4E in iRapKO MEF models; however, phosphorylation of eIF4E occurred in all Raptor knockout cells whether infected with virus or mock infected. Thus, we conclude that phosphorylation of eIF4E is not sufficient to overcome loss of mTORC1 activity in the Raptor knockout MEF system. Differences in the experimental results between groups may be due to approaches used to inhibit mTORC1. We use an inducible Raptor gene deletion system that results in a total loss of mTORC1 activity while other studies used small interfering RNA (siRNA) approaches and biochemical inhibitors, which may have different effects on cellular translation initiation for the duration of an experiment. For example, P70S6K and 4EBP compete for Raptor binding prior to phosphorylation by mTORC1 [13], and phosphorylation of 4EBP1 recovers from mTORC1 inhibition much more rapidly and effectively than p70S6K phosphorylation. Thus, treatment with an mTOR catalytic site inhibitor blocks phosphorylation for both species, but 4EBP is more resistant to mTOR inhibition because it recovers quickly [14]. This implies that studies that use transient pharmacologic inhibition of mTOR signaling may disproportionately inhibit p70S6K activity more than 4EBP activity. 

Our data also show that canonical markers of mTORC1 activation, phosphorylation of p70S6K and rpS6 have no impact on WNV growth and no effect on viral protein production for positive strand viruses, such as WNV. We used an MEF cell line with gene knockout of *S6K1* and *S6K2* with resulting loss of p70S6K activity. Based on these studies, S6K signaling downstream of mTORC1 is dispensable for WNV growth despite the established role of S6K in promoting protein translation [20]. This is important since p70S6K is a major, central signaling kinase, and these findings support two important conclusions. First, different mRNAs require different support from host translational signaling, and RNA viruses like WNV utilize specific host signals to support translation, while other host signals, such as S6K and rpS6, are dispensable for the virus. Second, the reason for decreased WNV growth in the Raptor knockout cells cannot be attributed to loss of central, signaling kinases since knockout of S6K had no effect on WNV growth or protein expression.

Conversely, we found that WNV growth and protein production were dependent on 4EBP expression and eIF4F complex formation. Without 4EBP expression to regulate the availability of eIF4E in host cells, we found a significant WNV growth defect and decreased viral protein production. This was an unexpected result as 4EBP is considered a negative regulator of eIF4E function, and loss of the negative regulator should result in a larger free pool of eIF4E. Instead, we found no change in total eIF4E expression and found that knockout of 4EBP decreased viral growth and viral protein synthesis in both a single-step and a multi-step viral growth curve. We propose several possible explanations for these findings. Interactions between mTORC1, 4EBP and eIF4E may result in different localization of the eIF4E pool to support translation events at different cellular sites such as cytoplasmic or membrane-bound ribosomes. Alternatively, the results with 4EBP may be complicated by involvement with changes in host translation events of innate immune transcripts. In fact, translation of specific subsets of mRNA defined by the 5′ UTR structure may be partially determined by specific interactions with eIF4E and 4EBP [38,44,45,46].

Using multi-step WNV growth curves, we also found that Raptor knockout MEF cells exhibited a more profound effect on WNV growth (Figure 2A) when compared to the WNV multi-step growth curve in 4EBP knockout cells (Figure 6B). These data imply that 4EBP may be an important output of control for WNV-induced mTORC1 activity, but may not be the only outcome of mTORC1 activity. Our prior data show that WNV activates mTORC1 activity independent of autophagy and cell cycle [8,31]. Thus, the loss of mTORC1-dependent phosphorylation and inhibition of 4EBP has a more profound effect on WNV growth than loss of 4EBP. This is most likely due to decreased availability of eIF4E in the Raptor knockout system, especially as infection progresses. This potential mechanism is supported by the WNV multi-step growth curve in Raptor knockout MEF cells. While limiting eIF4E is available, WNV growth occurs early in Raptor knockout MEFs. However, as infection continues, sequestration of eIF4E by unphosphorylated 4EBP results in a quick plateau and then fall in WNV growth. This effect is even more profound following CHIKV infection, which is likely due to virus reliance on eIF4E binding for both genomic and subgenomic RNA translation. 

**Figure 8:**
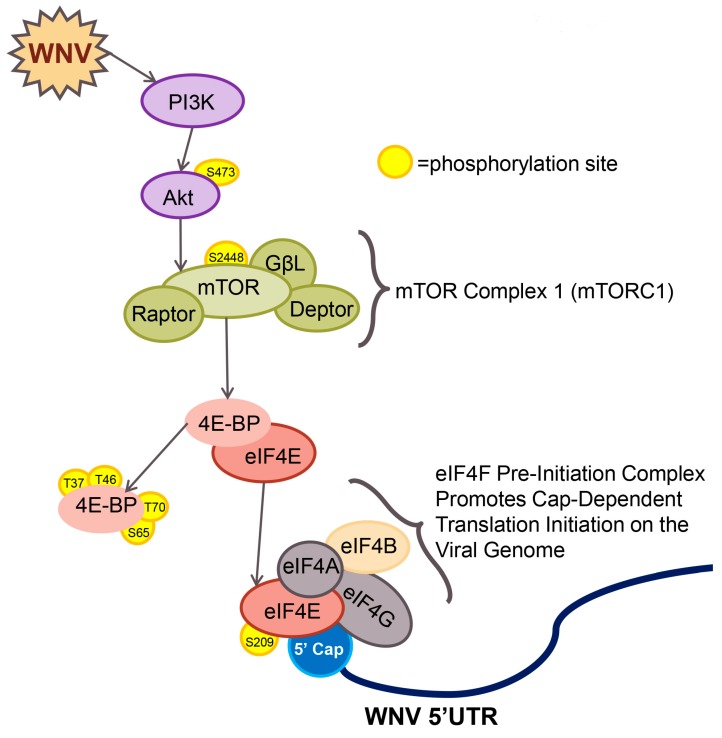
Working model for WNV activation of mTOR and the effect on viral RNA translation initiation events. WNV infection of host cells leads to activation of mTORC1 resulting in increased phosphorylation of the translation-initiation repressor 4EBP and dissociation from eIF4E. Free eIF4E binds to the 5′ cap on WNV genomic RNA and assembles the eIF4F pre-initiation complex required for cap-dependent translation of viral RNA, resulting in viral protein expression and viral growth.

Since deletion of 4EBP in our studies is not equivalent to preventing mTORC1-dependent phosphorylation of 4EBP, we evaluated the role of direct interactions between eIF4E and eIF4G at the cap using the 4EGI-1 inhibitor. We found that eIF4F complex formation supports (+) strand RNA virus growth and protein production in a dose-dependent fashion. Thus, we conclude that the mTORC1, 4EBP and eIF4E pathway supports viral protein production and viral growth. Based on our work to date, we propose a model for WNV-induced changes in host cell translation initiation signaling (Figure 8). 

Our observations suggest that the loss of mTORC1 impacts WNV replication at the level of translation, but not genomic replication and that these are two distinct, but linked processes in the flavivirus replication cycle. As the (+) sense RNA is the template for both genomic packaging and viral RNA translation, viral (+) sense RNA must be actively recruited for each function independently, as these disparate functions cannot occur concurrently on the same template. Based on our experimental observations, the deletion of Raptor and inactivation of mTORC1 may be impacting WNV growth in two ways. Deletion of mTORC1 activity may inhibit recruitment of the 40S pre-initiation complex and initiation of (+) sense RNA scanning or loss of mTORC1 cap-dependent initiation signals may result in a reduced pool of viral RNA in heavy polysomes. The polysome recruitment model would provide a definitive competitive advantage for viral RNA translation in a cellular environment in which viral RNA actually represents the minority of total RNAs species. At early stages of WNV infection, a single viral RNA in a heavy polysome could produce more functional protein than the equivalent genome load when limited to monosomes under conditions of reduced translation initiation. Further studies will be necessary to determine if mTORC1 activity is driving the formation of polysomes on 5′-capped (+) sense RNA viruses, such was WNV, and if loss of polysome formation on the viral genome is the mechanistic basis for our observed growth defects in this model.

Maintenance of proteostasis via mTORC1/4EBP/eIF4F is a key point of disruption in diverse disease mechanisms; whether it be virally induced, oncogenic or neurodegenerative [47,48,49,50,51,52,53]. Many of these pathogenic states are marked by the hyperactivation of Akt and mTORC1 and the downstream effectors S6K and 4EBPs [26,47,49,51,54,55,56]. The initiation of canonical cap-dependent translation depends on eIF4E and eIF4F complex formation, making the mTORC1/4EBP/eIF4E signaling cascade a potential target for future therapeutics for diverse viral infections [26,48,57]. This approach is aided by the development of new technologies, such as transcriptome-wide analysis, which has allowed researchers to probe the requirement for different parts of the eIF4F complex to translate RNAs with specific sequences or structures in the 5′ UTR [58]. 

We used EMCV as a control in our studies for cap-dependent virus translation since this virus uses an IRES. Prior work has shown that 4EGI may still inhibit EMCV and other IRES-controlled RNA viruses despite the fact that the mechanism of 4EGI-inhibition is thought to be specific to cap-binding events [41]. However, our data show that EMCV viral growth is largely resistant to the effects of the 4EGI inhibitor, but EMCV may exhibit decreased growth at higher doses of 4EGI-1 due to cell toxicity. We believe that our studies add to the existing literature on this topic since we used live virus infections and assayed viral titer, while prior studies have used reporter systems. These data may imply that other elements in the RNA genome of EMCV may be required to support IRES-independent activity to escape the effects of the 4EGI inhibitor. Additional studies with viruses that utilize different initiation strategies are required to understand the complex interactions between *cis*-acting RNA elements with translation initiation events for RNA viruses.

There are important weaknesses of the current study that must be taken into account. First, our studies as reported do not directly assay for RNA translation events. The logical extension of this work is to utilize new technologies such as ribosome profiling and other technical approaches to evaluate the effect of specific host signaling events during viral infection on viral and host RNA translation directly. Second, the effect of the 4EGI-1 inhibitor on the eIF4F complex may occur distal from the cap by competing with 4EBP. Thus, the effect of the 4EGI-1 inhibitor may be related to the viral requirement for 4EBP activity. This is an important consideration in these studies, and future studies defining the effect of 4EGI-1 on cap formation in vivo are required to determine the effect of this inhibitor on eIF4F formation. 

We used CHIKV in several experiments as an additional 5′ cap-dependent RNA virus control to show that the effects of cellular manipulation on viral growth are not specific to just WNV. It is important to note that many of the features of CHIKV growth following knockout of mTORC1 signaling are very different from WNV. For example, CHIKV viral growth and protein production are virtually nonexistent in Raptor knockout cells, while WNV seems to grow at a reduced rate with reduced protein production. It is important to note that CHIKV has different requirements for cap-dependent translation since the structural proteins are expressed from a subgenomic transcript. Thus, it is plausible that CHIKV has different interactions with specific host-translational events, since CHIKV growth in our system exhibits increased sensitivity to mTORC1-dependent signaling. Additional studies examining the interactions between 5′-capped RNA viruses and host translational control systems will lead to novel insight into translational control mechanisms and provide novel insights into potential broad-spectrum, host-targeted therapies that specifically inhibit viral translation while protecting host immune regulation.
